# 增加强度预处理单倍体造血干细胞移植治疗10例HLA供者特异性抗体（DSA）强阳性血液病患者临床观察

**DOI:** 10.3760/cma.j.issn.0253-2727.2023.08.007

**Published:** 2023-08

**Authors:** 慧霞 刘, 道林 魏, 珊 邵, 瑛 蒋, 肃 李, 骏 朱, 椿 王, 初娴 赵

**Affiliations:** 上海闸新中西医结合医院血液科，上海 200435 Department of Hematology, Shanghai Zhaxin Traditional Chinese & Western Medicine Hospital，Shanghai 200435, China

**Keywords:** 单倍体造血干细胞移植, 预处理, 供者特异性抗体, Haploidentical hematopoietic stem cell transplantation, Conditioning regimen, Donor specific antibodies

## Abstract

**目的:**

探讨增加强度预处理单倍体造血干细胞移植（haplo-HSCT）对于HLA供者特异性抗体（DSA）强阳性血液病患者的疗效。

**方法:**

纳入2019年5月1日至2022年9月1日在上海闸新中西医结合医院血液科接受增加强度预处理haplo-HSCT的DSA平均荧光强度（MFI值）≥10 000的10例血液病患者，对其临床资料进行回顾性分析。

**结果:**

①10例患者中男3例、女7例，中位年龄为53.5（36～64）岁；急性髓系白血病（AML）3例，骨髓增生异常综合征（MDS）2例，慢性粒-单核细胞白血病（CMML）2例，慢性髓性白血病加速期（CML-AP）2例，原发性骨髓纤维化（PMF）1例。②接受氟达拉滨（Flu）/白消安（Bu）为主联合全身放射治疗（TBI）/环磷酰胺（CTX）预处理方案。③移植前DSA中位MFI值为15 999（10 210～23 417），移植后第7天DSA中位MFI值为10 787（0～22 720）。④8例患者获得粒细胞和血小板植入，中位粒细胞植入时间为14（10～16）d，中位血小板植入时间为18（14～20）d。中位随访12.5（1.5～27）个月，原发植入失败、移植物植入功能不良患者各1例，7例患者DSA转为阴性且处于无进展生存状态。

**结论:**

增加强度预处理haplo-HSCT能够有效降低DSA强阳性血液病患者DSA强度，获得较好的近期疗效。

## Abstract (en)

**Objective:**

To explore the efficacy of immunosuppression intensified conditioning regimen in patients who have strongly positive donor-specific Anti-HLA antibodies（DSAs）and received a haploidentical hematopoietic stem cell transplantation（haplo-HSCT）.

**Methods:**

Clinical data of 10 patients with strongly positive pretransplant DSAs（defined as MFI ≥10000）were retrospectively analyzed in this study. All of them received a haplo-HSCT in the Hematology Department of Shanghai Zhaxin Traditional Chinese & Western Medicine Hospital.

**Results:**

① Of all ten patients, three were males, and seven were females, with a median age of 53.5（36−64）years. Of the 10 patients, three were diagnosed with acute myeloid leukemia, two were myelodysplastic syndromes（MDS）, two were chronic myelomonocytic leukemia（CMML）, two were in an accelerated phase of chronic myeloid leukemia（CML-AP）, and one was primary myelofibrosis（PMF）. ② Conditioning regimen consisted of fludarabine（Flu）/busulfan（Bu）combined with whole-body irradiation（TBI）/cyclophosphamide（Cy）. ③ On the seventh day after transplantation, the median pretransplant DSA level was MFI 15 999（10 210−23 417）and 10 787（0−22 720）. ④ Eight patients acquired hematopoietic reconstitution; the median time of neutrophil engraftment was 14（10−16）days; and 18（14−20）days for platelet engraftment. After a median follow-up of 12.5（1.5−27）months, primary graft failure was found in one patient and another with poor graft function. Seven patients remained in a disease remission state, and all were DSA-negative.

**Conclusion:**

An intensified immunosuppression conditioning regimen can efficiently decrease the level of donor-specific anti-HLA antibodies（DSAs）, leading to good short-term efficacy.

近年来随着造血干细胞移植技术的进步，单倍体造血干细胞移植（haplo-HSCT）可获得与HLA全相合异基因造血干细胞移植相似的疗效[1]，也使得更多患者有机会接受造血干细胞移植。然而，2.4％～15％的原发性植入失败（PGF）发生率仍然是导致haplo-HSCT患者死亡的主要原因之一[2]。Haplo-HSCT患者体内存在针对供者特异性抗体（donor specific antibody, DSA）是导致PGF的重要原因[3]。为了克服DSA对植入的不利影响，国内外各大移植中心对DSA检测及脱敏治疗进行了多种探索。欧洲血液骨髓移植学会（EBMT）2018年发表的相关指南推荐了多种DSA脱敏治疗方案[4]，但治疗周期均较长且难以评价疗效[5]–[7]。本中心近年来采用增加强度预处理haplo-HSCT治疗10例DSA强阳性血液病患者获得较好疗效。

## 病例与方法

一、病例资料

纳入2019年5月1日至2022年9月1日期间在本中心接受增加强度预处理haplo-HSCT的10例DSA强阳性血液病患者，对其临床资料进行回顾性研究。全部10例患者中男3例、女7例，中位年龄为53.5（36～64）岁。急性髓系白血病（AML）3例，骨髓增生异常综合征（MDS）2例，慢性粒-单核细胞白血病（CMML）2例，慢性髓性白血病加速期（CML-AP）2例，原发性骨髓纤维化（PMF）1例。基线资料见表1。

**表1:** 10例供者特异性抗体（DSA）强阳性血液病患者一般资料及单倍体造血干细胞移植前疾病状态

例号	性别	年龄（岁）	疾病诊断	染色体核型	融合基因/基因突变	疾病状态	FCM-MRD（%）
1	男	55	AML	正常	FLT3-ITD	CR	<0.01
2	女	55	CMML	46,XX;t（3;3）	SF3B1	NR	30
3	女	64	MDS	46,XX;del（20）（q13.1）	TP53	PR	5
4	男	63	MDS	正常	无	NR	9.5
5	女	53	PMF	47,XX,+8[8]	JAK2	NR	1
6	女	51	AML	46,XX;t（11;19）（q23;p13）	MLL/ELL	CR	<0.01
7	女	41	CMML	正常	无	NR	8
8	女	36	AML	46,XX,+5（q22）	FLT3-ITD	NR	62
9	男	54	CML-AP	46-47,XY,+8,+16,Ph	BCR-ABL	NR	18
10	女	49	CML-AP	44-46,XX	BCR-ABL	NR	6

**注** AML：急性髓系白血病；CMML：慢性粒-单核细胞白血病；MDS：骨髓增生异常综合征；PMF：原发性骨髓纤维化；CML-AP：慢性髓性白血病加速期；CR：完全缓解；PR：部分缓解；NR：未缓解；FCM-MRD：流式细胞术检测微量残留病

二、HLA抗体检测

所有患者均在移植前30～14 d和移植后7、14、28 d进行HLA抗体检测。HLA抗体检测采用免疫磁珠液相芯片技术（Luminex法），抗体强度水平以平均荧光强度（MFI值）表示。

三、增加强度预处理方案

氟达拉滨（Flu）30 mg·m^−2^·d^−1^（−6 d～−2 d）；白消安（Bu）3.2 mg·kg^−1^·d^−1^（−6 d～−4 d）；全身放射治疗（TBI）3 Gy，−8 d；环磷酰胺（CTX）30 mg·kg^−1^·d^−1^（−3 d、−2 d）；根据疾病类型及既往化疗用药情况，加用阿糖胞苷（Ara-C）1～2 g·m^−2^·d^−1^，−6 d～−2 d（总量5～15 g）。

四、DSA处理方案

利妥昔单抗375 mg/m^2^，−7 d；静脉注射免疫球蛋白0.4 g·kg^−1^·d^−1^，−6 d～−2 d。

五、GVHD预防

采用兔抗人胸腺细胞免疫球蛋白（rATG）联合他克莫司（FK-506）加短程甲氨蝶呤（MTX）的方案。rATG 2.5 mg·kg^−1^·d^−1^，移植前疾病完全缓解（CR）的患者应用4 d（−5 d～−2 d），未缓解（NR）的患者应用3 d（−4 d～−2 d）。他克莫司0.02 mg·kg^−1^·d^−1^，移植前2 d开始静脉滴注，维持药物浓度5～10 ng/ml；MTX于移植后第1、3、6天应用，剂量分别为15、10、10 mg/m^2^。

六、造血干细胞回输

造血干细胞回输日上午输注1份HLA低分辨至少4/6相合的脐带血，下午输注供者外周血造血干细胞，要求有核细胞计数≥10×10^8^/kg，CD34^+^细胞计数≥4×10^6^/kg。

七、定义及标准

根据EBMT指南[4]及文献[8]–[9]，本研究将MFI值≥500判定为DSA阳性，≥10000判定为强阳性。粒细胞植入定义为中性粒细胞绝对计数（ANC）>0.5×10^9^/L连续3 d，血小板植入定义为PLT>20×10^9^/L连续7 d且脱离血小板输注。PGF是指移植后28 d时未获得粒细胞和血小板植入，继发性植入失败是指在已经获得植入的基础上再次出现三系中至少两系的血细胞计数下降或丢失供患者嵌合状态[9]。植入功能不良定义为移植28 d以后两系或三系细胞计数未达到植活标准持续2周以上，骨髓检查提示骨髓增生低下，原发病处于缓解状态，细胞为完全供者嵌合，而无严重移植物抗宿主病（GVHD）和复发[10]。急性GVHD采用Glucksberg分级系统，依据为皮疹所占体表面积以及胆红素水平以及腹泻量。慢性GVHD按西雅图诊断标准以及NIH分级系统进行各脏器分级及分度。疗效分为完全缓解、部分缓解、无效[11]–[12]。

八、随访

根据患者CD3^+^淋巴细胞短串联重复序列进行分析或性染色体FISH分析（若供患者性别不同）判断移植后嵌合状态[13]–[14]。根据移植后嵌合状态以及微量残留病（MRD）检测情况确定随访的时间点。移植后2周开始随访，每2周1次直到供者细胞完全嵌合，随后根据移植前原发病状态制定随访计划，低危患者（移植前CR且MRD检测阴性）每月1次随访1年，此后每2个月随访1次至移植后2年；高危患者（不符合低危标准）每2周随访1次至移植后3个月，此后每月1次随访1年，此后每2个月随访1次至2年。随访截止为2022年9月1日。

九、统计学处理：采用SPSS 22.0软件，选用自身配对*t*检验的方法，对移植前后DSA的MFI值变化进行两两比较，以*P*<0.05为差异具有统计学意义。

## 结果

一、移植特征

10例患者的供者均为子女，移植物为外周血造血干细胞。供、患者ABO血型相合6例，主要不合1例、次要不合1例、主次均不合2例。采用ECOG体能状态评分和造血干细胞移植前合并症指数（HCT-CI）行移植前评估，5例患者ECOG评分为3分，2例患者HCT-CI评分为3分。单个核细胞（MNC）中位输注量为20.1（12.7～29.1）×10^8^/kg，CD34^+^细胞中位输注量为15.1（4.4～22.5）×10^6^/kg。移植相关资料见表2。

**表2:** 10例供者特异性抗体（DSA）强阳性血液病患者单倍体造血干细胞移植相关资料

例号	ECOG	HCT-CI	供患者关系	供者年龄（岁）	HLA配型	供患者血型	输血依赖	MNC（×10^8^/kg）	CD34^+^细胞（×10^6^/kg）
1	2	1	女供父	19	6/12	A供A	否	19.1	14.9
2	3	3	子供母	36	6/12	B供B	是	28.9	22.3
3	2	2	女供母	39	7/12	AB供B	是	13.6	4.4
4	3	3	子供父	39	6/12	A供B	是	21.1	17.3
5	2	2	女供母	29	8/12	B供B	是	24.9	17.5
6	2	2	子供母	32	6/12	O供O	否	29.1	22.5
7	3	2	子供母	17	6/12	B供AB	是	14.9	11.6
8	3	1	子供母	12	6/12	A供B	是	12.7	9.8
9	3	2	子供父	25	7/12	B供B	是	18.1	15.2
10	2	1	子供母	27	7/12	A供A	是	21.2	12.3

**注** HCT-CI：造血干细胞移植前合并症指数；MNC：单个核细胞

二、DSA动态监测

10例患者移植前DSA的抗体类型包括仅针对Ⅰ类抗原或Ⅱ类抗原或者两类兼有的抗体。移植前DSA MFI值中位数为15 999（10 210～23 417）。预处理后患者体内DSA逐渐下降，移植后第7天的DSA中位MFI值为10787（0～22 720）。移植前与移植后第7、14、28天数据两两比较，DSA明显下降（*P*<0.003）。第28天有7例患者DSA转为阴性，3例患者DSA MFI值仍>500。例2针对DRB1*07:01位点的抗体MFI值在第7天上升至>20 000，第28天时HLA-Ⅱ类抗原三个位点仍为DSA强阳性。例5和例8移植前DSA MFI值均>20 000，经预处理后MFI值明显下降，DSA具体变化情况见表3。

**表3:** 10例供者特异性抗体（DSA）强阳性血液病患者移植前后DSA水平

例号	HLA位点（抗体类型）	平均荧光强度			
		移植前	移植后7 d	移植后14 d	移植后28 d
1	DRB1*11:01（II）	12 474	5 467	226	0
2	B*13:02（I）	13 298	0	0	0
	DRB1*07:01（II）	18 475	22 720	21 415	16 574
	DQ1B1*02:02（II）	16 614	14 069	12 584	11 245
	DPB1*17:01（II）	14 295	14 294	12 344	10 513
3	DQB1*06:02（II）	10 210	564	235	0
4	DRB1*09:01（II）	12 427	9 571	2 724	333
	DQB1*03:03（II）	15 966	18 643	3 629	754
5	C*03:04（I）	23 417	15 153	14 612	7 028
6	C*03:04（I）	16 032	519	234	0
7	A02:01（I）	19 815	15 410	2 500	421
	DRB1*12:02（II）	16 514	0	0	0
8	B*46:01（I）	13 521	8 724	3 564	0
	DQB1*03:03（II）	21 051	15 421	2 431	0
9	B*13:01（I）	14 284	845	245	0
10	DRB1*11:01（II）	17 143	12 007	645	0

三、植入与嵌合状态

除了例2和例4，其余8例患者粒细胞与血小板均成功植入，中位粒细胞植入时间为14（10～16）d，中位血小板植入时间为18（14～20）d。移植后第28天嵌合度分析，8例患者中位嵌合率为98.7％（97.0％～99.8％）。例2预处理后DSA持续强阳性，移植后第14、28天嵌合度分别为85％、18％，判定为PGF，于首次移植后第40天行二次造血干细胞移植（未更换供者），第二次移植预处理后再次检测DSA MFI值均明显下降（MFI<500），二次移植后15 d、20 d分别获得粒细胞植入、血小板植入，移植后17天骨髓嵌合度为100％。例4为老年MDS患者，移植后14、28天嵌合度分别为98.6％、99.5％，但外周血象未恢复，考虑为移植物植入功能不良，移植后第45天因感染死亡。10例患者移植治疗结果详见表4。

**表4:** 10例供者特异性抗体（DSA）强阳性患者增加强度预处理单倍体造血干细胞移植结果

例号	诊断	移植前疾病状态	植入	粒细胞植入（d）	血小板植入（d）	移植后疾病状态	急性GVHD	慢性GVHD	随访时间（月）	转归
1	AML	CR	是	+15	+14	CR	皮肤Ⅱ度，总体Ⅰ度	无	10	存活
2^a^	CMML	NR	是	+15	+20	CR	皮肤Ⅳ度肝脏Ⅳ度肠道Ⅲ度，总体Ⅳ度	NA	3	死亡
3	MDS	PR	是	+10	+16	CR	无	口腔溃疡	15	存活
4	MDS	NR	否	/	/	NR	无	NA	1.5	死亡
5	PMF	NR	是	+12	+19	CR	无	无	13	存活
6	AML	CR	是	+14	+17	CR	皮肤Ⅱ度，总体Ⅰ度	无	12	存活
7	CMML	NR	是	+14	+18	CR	无	口腔溃疡	27	存活
8	AML	NR	是	+13	+19	CR	皮肤Ⅱ度，总体Ⅰ度	无	25	存活
9	CML-AP	NR	是	+15	+18	CR	皮肤Ⅳ度肝脏Ⅳ度肠道Ⅳ度，总体Ⅳ度	NA	2	死亡
10	CML-AP	NR	是	+16	+20	CR	皮肤Ⅱ度，总体Ⅰ度	无	24	存活

**注** AML：急性髓系白血病；CMML：慢性粒-单核细胞白血病；MDS：骨髓增生异常综合征；PMF：原发性骨髓纤维化；CML-AP：慢性髓性白血病加速期；GVHD：移植物抗宿主病；CR：完全缓解；PR：部分缓解；NR：未缓解；^a^ 例2首次移植原发植入失败，二次移植成功

四、生存状况

随访至2022年9月1日，中位随访时间为12.5（1.5～27）个月。7例（70％）患者均为无进展生存状态（包括5例移植前NR患者）。3例患者死亡。

五、GVHD

10例患者中，4例无明显急慢性GVHD表现。4例患者表现为Ⅱ度皮肤GVHD，糖皮质激素治疗后好转，3例患者发生局限慢性GVHD，主要表现为口腔溃疡，NIH评分均为1分。2例患者发生Ⅳ级急性GVHD，累及皮肤、肝脏以及胃肠道。其中例2于第二次移植后2个月死于Ⅳ度肝脏GVHD。例9抗GVHD治疗后，皮疹及黄疸消退，腹泻量减少50％，考虑治疗有效，移植后2个月死于肠道感染。

## 讨论

HLA DSA是患者体内存在的针对HLA错配供者的循环抗体，其靶向供者的HLA-Ⅰ/HLA-Ⅱ类抗原。在实体器官移植中，这些预先形成的循环抗体可能导致超急性移植器官排斥反应。随着haplo-HSCT病例逐年增多，由抗体介导的干细胞植入失败/延迟问题受到重视。MDACC研究小组在2009年报道的haplo-HSCT病例中，5例DSA阳性患者，3例发生PGF，提示haplo-HSCT患者的DSA与PGF存在关联[15]。随后该小组回顾分析了122例单倍体移植病例（DSA阳性患者22例），阳性组与阴性组PGF的发生率分别为32％、4％（*P*<0.001）[16]。Spellman等[17]报道37例植入失败的患者，其中9例（24％）为DSA阳性。最近，一项荟萃分析[18]纳入15项研究共2436例接受haplo-HSCT或脐血造血干细胞移植的患者，DSA阳性患者PGF的发生风险增加7.47倍。DSA阳性在妊娠、多次输血、既往接受过器官移植患者中的发生率较高。目前，DSA检测手段包括补体依赖的细胞毒性试验（CDC）、流式细胞术、酶联免疫吸附试验（ELISA）和免疫磁珠液相芯片法（Luminex）。其中Luminex具有高特异性、高敏感性等优点，是美国组织相容性及免疫遗传学协会（ASHI）推荐方法。Luminex的DSA检测结果以MFI值的高低判断抗体的强弱。然而，各中心判断DSA阳性的MFI界值并不统一。Takanashi等[19]对386例患者进行回顾性分析，将MFI值≥1 000定义为DSA阳性。Ciurea等[20]发现植入失败患者移植前DSA平均MFI值为10 055，明显高于对照组（平均MFI值为2 065）。Chang等[9]在345例haplo-HSCT患者中观察到MFI值>10 000组具有较高的植入失败发生率。本研究，我们参照EBMT标准将MFI值≥500定义为DSA阳性，MFI≥10 000定义为强阳性。

基于DSA对供者干细胞植入的不良影响，干细胞回输前可应用脱敏方法降低DSA的抗体滴度。2018年EBMT共识指南中推荐了以下联合脱敏方案：①通过血浆置换或免疫吸附清除抗体；②应用静脉注射免疫球蛋白（IVIg）或应用供者HLA抗原（供者血小板或白膜层输注）中和体内抗体；③应用针对CD20^+^B淋巴细胞的单克隆抗体（利妥昔单抗）或者针对产生同种抗体的浆细胞的蛋白酶体抑制剂（硼替佐米）抑制抗体产生；④应用靶向终末补体蛋白C5的单克隆抗体，阻断补体级联反应。然而，此共识所依据的研究结果大多基于实体器官移植经验且多为单中心小型研究，缺乏大规模随机对照的前瞻性研究数据。

MDACC报道[20]的DSA脱敏方案如下：−25 d、−22 d、−19 d、−16 d各应用硼替佐米1次；−15 d、−13 d、−11 d行血浆置换；−10 d输注IVIg；−9 d予利妥昔单抗；−7 d ～−2 d行移植预处理，−1 d予以供者白膜层输注。研究入组48例DSA阳性患者，脱敏治疗组26例，治疗前DSA平均MFI值为4 887，治疗后为2 906，供者干细胞植入率为92.3％。提示进行有效的DSA脱敏治疗能成功纠正DSA阳性对植入的不利影响。

Ciurea等[21]采用的DSA脱敏方案：−15 d、−13 d、−11 d行血浆置换；−10 d予利妥昔单抗；−9 d输注IVIg；−7 d～−2 d行移植预处理；−1 d予以供者白膜层输注。DSA阳性患者37例，31例MFI值≤20 000的患者中性粒细胞植入率、非复发死亡率、总生存率与DSA阴性对照组相当，而MFI值>20 000的6例患者中4例植入失败，提示移植前DSA的抗体强度与PGF存在相关性，现有多药组合的脱敏方案仍不能改善MFI值>20 000患者的植入。

现有DSA联合脱敏方案历时长，用药繁琐，且需要用大量血浆进行抗体置换，对于亟需进行挽救治疗的患者而言，适用性差。移植前1 d行供者血小板或白膜层输注，增加一次供者采集操作。因此，亟待探索针对存在DSA阳性，尤其是高MFI值的DSA阳性患者的脱敏方案。

现有DSA脱敏方案中针对B淋巴细胞的抑制主要依靠利妥昔单抗和硼替佐米实现，显然达不到清除的目的。我们在以FluBu为主的预处理基础上加入TBI以及CTX，以期增强对淋巴细胞的清除作用，不仅着眼于对宿主残留T淋巴细胞的清除，同时加强对产生抗体的B淋巴细胞清除，再联合应用利妥昔单抗和IVIg，达到耗竭B淋巴细胞并封闭已产生的循环抗体的目的。本研究纳入10例患者，移植前DSA 中位MFI为15 999，其中2例患者MFI值>20 000。本组病例移植后第28天10例患者中有9例获得中性粒细胞及血小板植入（例2二次移植）。我们的研究结果与国外数据相近。Spellman等[17]报道了37例DSA阳性患者，脱敏前DSA平均MFI值为10 198，移植后28 d粒细胞累积植入率为75.7％，血小板累积植入率为50％。本研究中我们观察到DSA的脱敏疗效与干细胞植入存在相关性。本研究中例2患者首次移植接受增加强度预处理后，第28 d体内仍有针对HLA-Ⅱ类抗原的三个位点的DSA 且MFI值均>10 000。临床上，患者骨髓嵌合度低下造血功能不能恢复，判定为PGF，40 d后行同一供者二次移植，预处理后DSA MFI值下降，粒细胞及血小板成功植入，也提示DSA强度下降有利于造血干细胞植入。

综上，我们采用增加强度预处理haplo-HSCT治疗10例DSA强阳性血液病患者，显示出良好的近期疗效。本方案无需行血浆置换，不增加供者采血次数，脱敏治疗时间短，能为亟需行挽救性移植的患者争取治疗时机。本研究病例数不多且其中8例患者为难治/复发，移植后随访时间也较短，以上结论尚需前瞻性多中心随机对照研究证实。
